# Metformin Attenuates Osteoporosis in Diabetic Patients with Carcinoma in Situ: A Nationwide, Retrospective, Matched-Cohort Study in Taiwan

**DOI:** 10.3390/jcm9092839

**Published:** 2020-09-02

**Authors:** Chieh-Hua Lu, Chi-Hsiang Chung, Feng-Chih Kuo, Kuan-Chan Chen, Chia-Hao Chang, Chih-Chun Kuo, Chien-Hsing Lee, Sheng-Chiang Su, Jhih-Syuan Liu, Fu-Huang Lin, Chang-Huei Tsao, Po-Shiuan Hsieh, Yi-Jen Hung, Chang-Hsun Hsieh, Wu-Chien Chien

**Affiliations:** 1 Department of Internal Medicine, Division of Endocrinology and Metabolism, Tri-Service General Hospital, School of Medicine, National Defense Medical Center, Taipei 11490, Taiwan; undeca2001@gmail.com (C.-H.L.); shoummie@hotmail.com (F.-C.K.); kuanchanndmc@gmail.com (K.-C.C.); kittyrvyi@gmail.com (C.-H.C.); lg1001010@gmail.com (C.-C.K.); doc10383@gmail.com (C.-H.L.); shiyuan71@yahoo.com.tw (S.-C.S.); ajleonn21@hotmail.com.tw (J.-S.L.); metahung@yahoo.com (Y.-J.H.); 2 Department of Medical Research, National Defense Medical Center, Taipei 11490, Taiwan; pshsieh@hotmail.com; 3 School of Public Health, National Defense Medical Center, Taipei 11490, Taiwan; g694810042@gmail.com (C.-H.C.); noldling@ms10.hinet.net (F.-H.L.); 4 Taiwanese Injury Prevention and Safety Promotion Association, Taipei 10048, Taiwan; 5 Department of Medical Research, Tri-Service General Hospital, National Defense Medical Center, Taipei 11490, Taiwan; changhuei@mail.ndmctsgh.edu.tw; 6 Department of Microbiology & Immunology, National Defense Medical Center, Taipei 11490, Taiwan; 7 Department of Physiology and Biophysics, National Defense Medical Center, Taipei 11490, Taiwan; 8 Institute of Preventive Medicine, National Defense Medical Center, Taipei 11490, Taiwan

**Keywords:** osteoporosis, diabetes, metformin, carcinoma in situ, National Health Insurance Research Database

## Abstract

Patients with diabetes are at increased risk of cancer development and osteoporosis. Metformin is an effective agent for diabetes management. Epidemiological studies have identified an association between metformin use and cancer prevention. This article outlines the potential for metformin to attenuate the rate of osteoporosis in diabetic patients with carcinoma in situ (CIS). From the National Health Insurance Research Database of Taiwan, 7827 patients with diabetes with CIS who were receiving metformin therapy were selected, along with 23,481 patients as 1:3 sex-, age- and index year-matched controls, who were not receiving metformin therapy. A Cox proportional hazard analysis was used to compare the rate of osteoporosis during an average of 15-year follow-up. Of the subjects who were enrolled, 801 (2.56%) had osteoporosis, including 168 from the metformin group (2.15%) and 633 from the without metformin group (2.70%). The metformin group presented a lower rate of osteoporosis at the end of follow-up (*p* = 0.009). The Cox proportional hazard regression analysis revealed a lower rate of osteoporosis for the metformin group (adjusted hazard ratio of 0.820; 95% confidence interval = 0.691–0.972, *p* = 0.022). Diabetic patients with CIS under metformin therapy presented lower osteoporosis rate than those who were not receiving metformin therapy.

## 1. Introduction

Patients with diabetes are at increased risk of cancer development [1] and associated with an increased risk of fragility fractures compared to the general population [2]. Type 2 diabetes mellitus (T2DM) and cancer share many risk factors, such as age, obesity, diet and physical inactivity [3]. The possible mechanisms for a direct link between T2DM and cancer include hyperinsulinemia [4], hyperglycemia [5] and inflammation [6,7].

Diabetes and cancer are clinical risk factors used for fragility fracture probability assessments across the general population [8,9]. Osteoporosis is a skeletal disorder that is characterized by low bone mass and compromised bone strength [10]. T2DM may affect bone metabolism and leads to osteoporosis [11] and cancer may affect both, through the direct effects of cancer cells on the skeleton and the deleterious effects of cancer-specific therapies on bone cells [9].

Metformin is an effective agent for T2DM management [12]. Furthermore, epidemiological studies have identified an association between metformin use and cancer prevention [13]. Fractures are a clinically important consequence of osteoporosis and result not only in disabilities, but also in excess mortality; however, the pathogenic mechanisms that underlie the relationship between osteoporosis, diabetes and cancer remain incompletely understood. The aim of this study was to determine the potential for metformin to attenuate the rate of osteoporosis in diabetic patients with carcinoma in situ using data from the Taiwan National Health Insurance Research Database (NHIRD), which is a nationwide health insurance database.

## 2. Materials and Methods

### 2.1. Data Sources

Our study used data from the NHIRD to investigate whether metformin therapy in diabetic patients with carcinoma in situ could lower osteoporosis rates, compared to a group of individuals who were not receiving metformin, over a 15-year period, from the outpatient Longitudinal Health Insurance Database (LHID) in Taiwan (2000–2015). The National Health Insurance (NHI) Program was launched in Taiwan in 1995, and as of June 2009, it included contracts with 97% of the medical providers in Taiwan, with approximately 23 million beneficiaries or more than 99% of the entire Taiwan population [14]. The NHIRD uses International Classification of Diseases, 9th Revision, Clinical Modification (ICD-9-CM) codes to record diagnoses [15]. All diagnoses of T2DM, carcinoma in situ, and osteoporosis were made by a board-certified medical specialist. The Bureau of NHI randomly reviews the records of 1 in 100 ambulatory care visits and 1 in 20 in-patient claims, to verify the accuracy of the diagnoses [16]. Several studies have demonstrated the accuracy and validity of the diagnoses in the NHIRD [17,18].

### 2.2. Study Design and Sampled Participants

Our study was a retrospective matched-cohort design. Patients with diagnosed T2DM, carcinoma in situ, and osteoporosis were selected from 1 January 2000 to 31 December 2015, according to ICD-9-CM 230.XX-234.XX (carcinoma in situ), ICD-9-CM 733.XX (osteoporosis) and ICD-9-CM 250.XX (T2DM). Furthermore, each enrolled patient was required to have made at least three outpatient visits within the study period, according to these ICD-9-CM codes whether or not they were receiving metformin therapy. Patients with osteoporosis diagnoses before 2000 and those less than 18 years of age were excluded. Furthermore, patients with diabetes who received thiazolidinedione or canagliflozin therapy were also excluded.

The covariates included Charlson comorbidity index (CCI), T2DM, sex, age, geographical area of residence (north, center, south and east of Taiwan) and urbanization level of residence (level 1 to 4). The urbanization level of residence was defined according to the population and various indicators of the level of development. Level 1 was defined as a population >1,250,000 and a specific economic, cultural, metropolitan and political development designation. Level 2 was defined as a population between 500,000 and 1,249,999 that played an important role in the political system, culture and economy. Urbanization levels 3 and 4 were defined as either populations between 149,999 and 499,999 or <149,999, respectively [19].

### 2.3. Outcome Measures

All of the study participants were followed from the index date until the onset of osteoporosis, from the NHI program, before the end of 2015.

### 2.4. Statistical Analysis

All statistical analyses were performed using SPSS software version 22 for Windows (SPSS, Inc., Chicago, IL, USA). chi-squared and *t-*tests were used to evaluate the distributions of categorical and continuous variables, respectively. A regression analysis of multivariate Cox proportional hazards was used to determine the risk of osteoporosis under metformin therapy in patients with diabetes and carcinoma in situ. The statistical analyses were presented as hazard ratio (HR) with ninety-five percent confidence interval (CI). The difference in the risk of osteoporosis for patients with diabetes and carcinoma in situ, with or without metformin therapy, was estimated using the log rank test—Kaplan-Meier method. A two-tailed test *p*-value less than 0.05 was considered to indicate statistical significance.

### 2.5. Ethics

Our study was conducted in accordance with the Code of Ethics of the World Medical Association (Declaration of Helsinki). The Institutional Review Board of Tri-Service General Hospital (TSGH) approved our study and waived the need for individual written informed consent (TSGH IRB No.2-105-05-082).

## 3. Results

**Figure 1:**
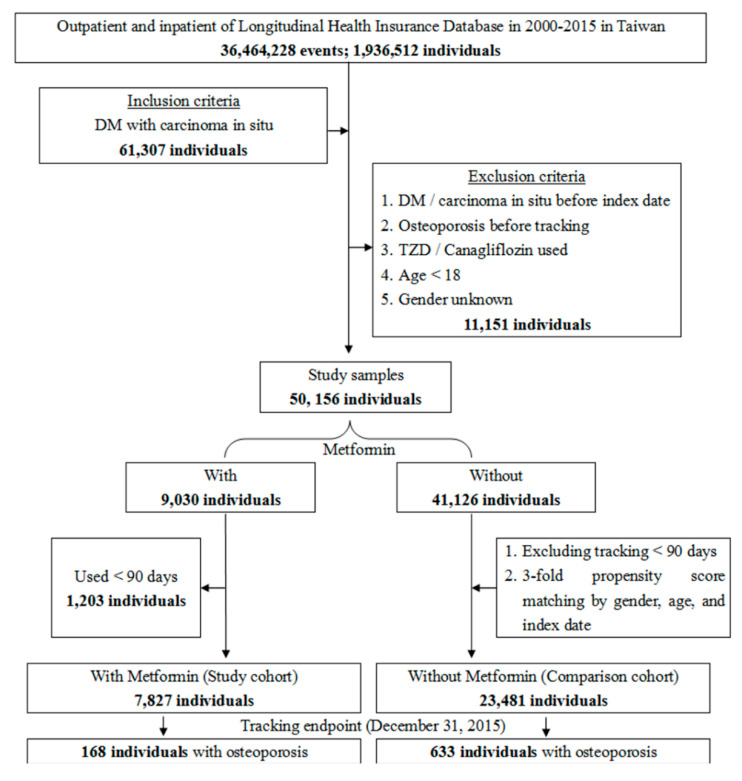
Flowchart of study patient selection from the National Health Insurance Research Database in Taiwan. DM—Diabetes mellitus: ICD-9-CM 250; Carcinoma in situ: ICD-9-CM 230–234; Osteoporosis: ICD-9-CM 733.0; Metformin: ≥90 days.

**Figure 2:**
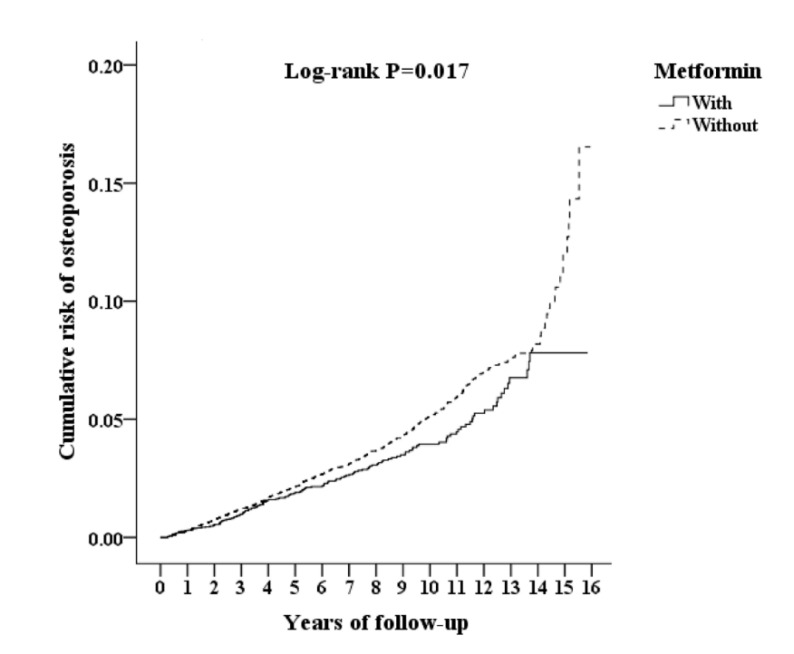
Kaplan-Meier analysis of cumulative risk of osteoporosis among patients with diabetes mellitus with carcinoma in situ aged 18 and over, stratified by metformin use and analyzed using a log-rank test.

Of the 61,307 enrolled patients, 11,151 were excluded and 50,156 patients with cancer and T2DM were included. Furthermore, 9030 of the included patients were receiving metformin therapy, of which 1203 patients had used metformin for less than 90 days and were excluded and 7827 were enrolled (case group). For the control group, we enrolled 41,126 individuals who were not receiving metformin therapy to represent the 1:3 sex-, age- and index year-matched control group and excluded 17,645 patients used metformin less than 90 days, for a final count of 23,481 subjects (control group) (Figure 1). Overall, the diabetic patients with carcinoma in situ under metformin therapy presented a lower rate of osteoporosis than those who were not receiving metformin (adjusted HR 0.820 (95% CI = 0.691–0.972, *p* = 0.022). Figure 2 shows the Kaplan-Meier analysis for the cumulative risk of osteoporosis in patient and control groups and the difference is statistically significant (log rank, *p* = 0.017).

The baseline sex, age, comorbidities, location, urbanization, level of care and income of the study subjects and controls is presented in Table 1. Of the 31,308 adult diabetic patients with carcinoma in situ, 13,284 (42.43%) were male, 12,928 (41.29%) were ≥60 year of age and the mean age was 55.95 ± 14.28 years (Table 1). There were no significant differences between the metformin and control groups in age distribution, sex, comorbidities and covariates, after propensity-score matching.

At the end of the follow-up period, 801 enrolled subjects (2.56%) had osteoporosis, including 168 from the metformin group (2.15%) and 633 from the without metformin group (2.70%), as shown in Table 2. The metformin group was associated with a lower rate of osteoporosis at the end of the follow-up (*p* = 0.009). There were no significant differences between the metformin and control groups in age distribution, sex, comorbidities and covariates at the end of the follow-up period.

The results of the Cox regression analysis of the factors associated with osteoporosis are shown in Table 3. The Cox proportional hazard regression analysis showed a lower rate of osteoporosis for patients receiving metformin therapy (adjusted hazard ratio of 0.820; 95% confidence interval = 0.691–0.972, *p* = 0.022).

For the subgroups in which osteoporosis factors were stratified by the variables listed in Table 4, the Cox regression analysis showed that the male, age < 60 years, better income, without catastrophic illness, unmarried, longer education, live in the higher urbanization level and care in hospital center were associated with a much lower osteoporosis rate.

## 4. Discussion

We found that diabetic patients with carcinoma in situ under metformin therapy presented lower osteoporosis rates than those who were not receiving metformin. The overall adjusted HR was 0.820 (*p* = 0.022), even after adjusting for comorbidities and other covariates. The Kaplan-Meier analysis revealed that the study subjects had a significantly lower 15-year risk of osteoporosis than the controls. This study is the first to indicate that diabetic patients with carcinoma in situ under metformin therapy have a lower osteoporosis risk in a nationwide, population-based study.

Prior research that has found that patients with diabetes have a relatively high risk of fracture [20], and one systematic review that showed that patients with diabetes have up to a three-fold greater fracture risk than the average person, with hip fracture being the largest [21]. Cancer is a major risk factor for osteoporosis, which is a common bone disease characterized by reduced bone mass and increased risk of fracture. Furthermore, these factors are associated both with the direct effects of cancer cells on the skeleton and the deleterious effects of cancer-specific therapies on bone cells [9]. One review article showed that certain key factors, osteoprotegerin (OPG)/ receptor activator of NF-κB ligand (RANKL)/receptor activator of NF-κB (RANK), underlie the molecular mechanism of osteoclastogenesis [22], and the anti-human RANKL monoclonal antibody has been successfully applied to the treatment of osteoporosis and cancer-related bone disorders [23,24].

Metformin is the preferred initial pharmacologic medicine for T2DM treatment, unless there are contraindications [25]. First-line metformin therapy has beneficial effects on HbA1C and weight [26] and may reduce the risk of cardiovascular events and death [27]. Patients with diabetes who received thiazolidinedione or canagliflozin therapy were excluded, due to the potential risk of osteoporosis [28]. Some studies have reported that older age, diabetes, and cancer under chemotherapy were associated with increased fracture risk, which was associated with elevated bone resorption [29,30].

Will metformin treatment reduce the incidence of osteoporosis in diabetic patients with carcinoma in situ? No study has investigated this in the past, and therefore, we used a cohort investigation to evaluate this issue. We found that the diabetic patients with carcinoma in situ under metformin therapy presented lower osteoporosis rates than those who were not receiving metformin. Evans and other scholars first proposed that metformin could reduce the cancer risk of patients with diabetes through epidemiological studies [31], in accordance with our results.

The impact of metformin on osteoporosis has been studied in the past. In vitro data on metformin suggest a protective effect on bones [32] and that metformin improves bone quality and decreases the risk of fracturs in patients with diabetes, in addition to improving glycemic control and insulin sensitivity [33]. Recent studies have shown that metformin can be osteogenic in vitro through the activation of AMP-activated protein kinase (AMPK), which results in osteoblastic cells differentiation, bone matrix synthesis and osteoblasts proliferation [34,35]. An in vivo study in mice showed that metformin enhances osteoblast proliferation and inhibits osteoclast differentiation to attenuate cancellous bone loss [36]. Furthermore, molecular research has found that metformin reduces RANKL and stimulates OPG expression in osteoblasts, which further inhibits osteoclast differentiation and prevents bone loss in ovariectomized rats [37]. Furthermore, metformin has been found to promote the proliferation of murine preosteoblasts by regulating the AMPK-mechanistic target of rapamycin 2 (mTOR2) and the AKT-mTORC1 signaling axis [38]. Additionally, metformin promotes the proliferation and differentiation of murine preosteoblasts by regulating the expression of sirtuin 6 (sirt6) and oct4 [39].

We therefore hypothesized that diabetic patients with carcinoma in situ who are on metformin therapy may present attenuated cancellous bone loss through metformin regulation of AMPK signaling in preosteoblasts, which reduces RANKL and stimulates OPG expression. Hence, metformin could be associated with reduced osteoporosis. Our findings are similar to many studies that have shown that females and older patients have a greater risk of osteoporosis. Clinical factors could be associated with those projected by demographic changes, with regards to age- and sex-specific risks [40]. Otherwise, the reasons why the subgroups that lived in higher urbanized areas and received therapy in hospital centers showed lower rates of osteoporosis are unknown and warrant further studies.

The present study has a few limitations. First, our study lacks analyses of disease duration, disease severity and patient parameters, such as body weight, BMI and waistline. Hip fracture risk is increased in patients with diabetes, whereas BMD is increased in patients with T2DM [41]. While the pathophysiological mechanism is only partially understood, a common complication may explain the increased fracture risk, whereas BMI may ameliorate the increased fracture risk in patients with T2DM [42]. Finally, a longer follow-up period may be necessary to clarify the osteoporosis risk for diabetic patients with carcinoma in situ.

In conclusion, diabetic patients with carcinoma in situ under metformin therapy presented a lower osteoporosis rate than those who were not receiving metformin therapy, and this effect may be attributed to the decreased levels of proinflammatory factors and the potential for metformin to modulate molecular pathways involved in cancer cell signaling and metabolism.

## Figures and Tables

**Table 1:** Characteristics of study in the baseline.

Metformin	Total		With		Without		*p*
Variables	*n*	%	*n*	%	*n*	%	
Total	31,308	-	7827	25.00	23,481	75.00	-
Gender	-	-	-	-	-	-	0.999
Male	13,284	42.43	3321	42.43	9963	42.43	-
Female	18,024	57.57	4506	57.57	13,518	57.57	-
Age (years)	55.95 ± 14.28		55.91 ± 14.36		55.96 ± 14.25		0.759
Age groups (years)	-	-	-	-	-	-	0.999
18–49	11,960	38.20	2990	38.20	8970	38.20	-
50–59	6420	20.51	1605	20.51	4815	20.51	-
≥60	12,928	41.29	3232	41.29	9696	41.29	-
Low-income	-	-	-	-	-	-	0.952
Without	30,998	99.01	7749	99.00	23,249	99.01	-
With	310	0.99	78	1.00	232	0.99	-
Catastrophic Illness	-	-	-	-	-	-	0.206
Without	25,374	81.05	6382	81.54	18,992	80.88	-
With	5934	18.95	1445	18.46	4489	19.12	-
Marital status	-	-	-	-	-	-	0.073
Without	12,841	41.02	3278	41.88	9563	40.73	-
With	18,467	58.98	4549	58.12	13,918	59.27	-
Education (years)	-	-	-	-	-	-	0.683
<12	13,692	43.73	3407	43.53	10,285	43.80	-
≥12	17,616	56.27	4420	56.47	13,196	56.20	-
CCI_R	0.61 ± 1.75		0.60 ± 1.70		0.61 ± 1.77		0.641
Season	-	-	-	-	-	-	0.866
Spring (Mar–May)	8070	25.78	2037	26.03	6033	25.69	-
Summer (Jun–Aug)	8425	26.91	2101	26.84	6324	26.93	-
Autumn (Sep–Nov)	7896	25.22	1982	25.32	5914	25.19	-
Winter (Dec–Feb)	6917	22.09	1707	21.81	5210	22.19	-
Location	-	-	-	-	-	-	0.659
Northern Taiwan	13,289	42.45	3335	42.61	9954	42.39	-
Middle Taiwan	8492	27.12	2150	27.47	6342	27.01	-
Southern Taiwan	8227	26.28	2017	25.77	6210	26.45	-
Eastern Taiwan	1253	4.00	315	4.02	938	3.99	-
Outlets islands	47	0.15	10	0.13	37	0.16	-
Urbanization level	-	-	-	-	-	-	0.877
1 (Highest)	12,423	39.68	3115	39.80	9308	39.64	-
2	14,419	46.06	3603	46.03	10,816	46.06	-
3	1346	4.30	324	4.14	1022	4.35	-
4 (Lowest)	3120	9.97	785	10.03	2335	9.94	-
Level of care	-	-	-	-	-	-	0.083
Hospital center	16,949	54.14	4319	55.18	12,630	53.79	-
Regional hospital	10,975	35.05	2668	34.09	8307	35.38	-
Local hospital	3384	10.81	840	10.73	2544	10.83	-

P—chi-squared/Fisher’s exact test on category variables and *t*-test on continue variables.

**Table 2:** Characteristics of study in the endpoint.

Metformin	Total		With		Without		*p*
Variables	*n*	%	*n*	%	*n*	%	
Total	31,308	-	7827	25.00	23,481	75.00	-
Osteoporosis	-	-	-	-	-	-	0.009
Without	30,507	97.44	7659	97.85	22,848	97.30	-
With	801	2.56	168	2.15	633	2.70	-
Gender	-	-	-	-	-	-	0.999
Male	13,284	42.43	3321	42.43	9963	42.43	-
Female	18,024	57.57	4506	57.57	13,518	57.57	-
Age (yrs)	61.37 ± 15.55		61.32 ± 15.60		61.39 ± 15.54		0.707
Age groups (yrs)	-	-	-	-	-	--	0.393
18–49	8106	25.89	2061	26.33	6045	25.74	-
50–59	6647	21.23	1625	20.76	5022	21.39	-
≥60	16,555	52.88	4141	52.91	12,414	52.87	-
Low-income	-	-	-	-	-	-	0.336
Without	30,936	98.81	7726	98.71	23,210	98.85	-
With	372	1.19	101	1.29	271	1.15	-
Catastrophic Illness	-	-	-	-	-	-	0.652
Without	22,517	71.92	5647	72.15	16,870	71.85	-
With	8791	28.08	2180	27.85	6611	28.15	-
Marital status	-	-	-	-	-	-	0.058
Without	12,846	41.03	3283	41.94	9563	40.73	-
With	18,462	58.97	4544	58.06	13,918	59.27	-
Education (years)	-	-	-	-	-	-	0.659
<12	13,687	43.72	3405	43.50	10,282	43.79	-
≥12	17,621	56.28	4422	56.50	13,199	56.21	-
CCI_R	1.96 ± 3.70		1.99 ± 3.76		1.95 ± 3.68		0.320
Season	-	-	-	-	-	-	0.486
Spring	7236	23.11	1782	22.77	5454	23.23	-
Summer	7995	25.54	2017	25.77	5978	25.46	-
Autumn	8780	28.04	2232	28.52	6548	27.89	-
Winter	7297	23.31	1796	22.95	5501	23.43	-
Location	-	-	-	-	-	-	0.499
Northern Taiwan	12,321	39.35	3067	39.18	9254	39.41	-
Middle Taiwan	9222	29.46	2340	29.90	6882	29.31	-
Southern Taiwan	8108	25.90	1993	25.46	6115	26.04	-
Eastern Taiwan	1548	4.94	399	5.10	1149	4.89	-
Outer islands	109	0.35	28	0.36	81	0.34	-
Urbanization level	-	-	-	-	-	-	0.727
1 (Highest)	10,135	32.37	2522	32.22	7613	32.42	-
2	14,118	45.09	3502	44.74	10,616	45.21	-
3	2131	6.81	537	6.86	1594	6.79	-
4 (Lowest)	4924	15.73	1266	16.17	3658	15.58	-
Level of care	-	-	-	-	-	-	0.590
Hospital center	12,771	40.79	3156	40.32	9615	40.95	-
Regional hospital	13,322	42.55	3340	42.67	9982	42.51	-
Local hospital	5215	16.66	1331	17.01	3884	16.54	-

P—chi-squared/Fisher’s exact test on category variables and *t*-test on continue variables.

**Table 3:** Factors of osteoporosis by using Cox regression.

Variables	Crude HR	95% CI	95% CI	*p*	Adjusted HR	95% CI	95% CI	*p*
Metformin	-	-	-	-	-	-	-	-
Without	1	-	-	-	1	-	-	-
With	0.813	0.686	0.964	0.017	0.820	0.691	0.972	0.022
Gender	-	-	-	-	-	-	-	-
Male	0.662	0.497	0.883	0.005	0.576	0.431	0.770	<0.001
Female	1	-	-	-	1	-	-	-
Age groups (yrs)	-	-	-	-	-	-	-	-
18–49	1	-	-	-	1	-	-	-
50–59	2.827	1.648	4.851	<0.001	3.113	1.813	5.345	<0.001
≥60	13.133	8.114	21.257	<0.001	15.456	9.533	25.059	<0.001
Low-income	-	-	-	-	-	-	-	-
Without	1	-	-	-	1	-	-	-
With	1.083	0.490	1.973	0.961	1.505	0.747	3.030	0.253
Catastrophic Illness		-	-	-	-	-	-	-
Without	1	-	-	-	1	-	-	-
With	0.521	0.426	0.638	<0.001	1.030	0.883	1.214	0.166
Marital status	-	-	-	-	-	-	-	-
Without	1	-	-	-	1	-	-	-
With	1.234	0.724	2.013	0.306	1.305	0.896	2.284	0.299
Education (years)	-	-	-	-	-	-	-	-
<12	1	-	-	-	1	-	-	-
≥12	0.903	0.512	1.894	0.376	0.865	0.483	1.881	0.425
CCI_R	0.892	0.860	0.925	<0.001	0.880	0.840	0.921	<0.001
Season	-	-	--	-	-	-	-	-
Spring	1	-	-	-	1	-	-	-
Summer	0.946	0.778	1.150	0.576	0.993	0.817	1.208	0.946
Autumn	0.785	0.644	0.957	0.017	0.769	0.653	0.971	0.024
Winter	1.008	0.827	1.229	0.938	1.013	0.831	1.235	0.898
Location	-	-	-	-	-	-	-	-
Northern Taiwan	1	-	-	-	Multicollinearity with urbanization level			
Middle Taiwan	1.318	1.111	1.563	0.002				
Southern Taiwan	1.151	0.958	1.383	0.113				
Eastern Taiwan	1.675	1.274	2.204	<0.001				
Islands outer of Taiwan	1.584	0.590	4.253	0.362				
Urbanization level	-	-	-	-	-	-	-	-
1 (Highest)	0.525	0.374	0.736	<0.001	0.560	0.420	0.827	0.002
2	0.707	0.591	0.846	<0.001	0.856	0.706	1.038	0.113
3	0.725	0.598	0.878	0.001	0.912	0.733	1.135	0.410
4 (Lowest)	1	-	-	-	1	-	-	-
Level of care	-	-	-	-	-	-	-	-
Hospital center	0.646	0.536	0.778	<0.001	0.712	0.594	0.853	<0.001
Regional hospital	0.670	0.560	0.800	<0.001	0.761	0.615	0.942	0.012
Local hospital	1	-	-	-	1	-	-	-

HR—hazard ratio; CI—confidence interval; Adjusted HR—adjusted variables listed in table.

**Table 4:** Factors of osteoporosis stratified by variables listed in table by using Cox regression.

Metformin	With			Without			Ratio	With vs. Without (Reference)			
Stratified	Events	PYs	Rate (per 10^5^ PYs)	Events	PYs	Rate (per 10^5^ PYs)		Adjusted HR	95% CI	95% CI	*p*
Total	168	83,045.71	202.30	633	254,028.18	249.18	0.812	0.820	0.691	0.972	0.022
Gender	-	-	-	-	-	-	-	-	-	-	-
Male	8	24,313.43	32.90	42	72,902.21	57.61	0.571	0.572	0.483	0.689	0.007
Female	160	58,732.28	272.42	591	181,125.97	326.29	0.835	0.843	0.710	1.006	0.054
Age groups (yrs)	-	-	-	-	-	-	-	-	-	-	-
18–49	1	16,823.43	5.94	16	48,608.18	32.92	0.181	0.180	0.150	0.218	<0.001
50–59	7	18,252.93	38.35	52	56,989.26	91.25	0.420	0.422	0.352	0.513	<0.001
≥60	160	47,969.35	333.55	565	148,430.74	380.65	0.876	0.886	0.746	1.072	0.241
Low-income	-	-	-	-	-	-	-	-	-	-	-
Without	166	82,125.32	202.13	626	251,334.43	249.07	0.812	0.818	0.682	0.954	0.019
With	2	920.40	217.30	7	2693.75	259.86	0.836	0.845	0.703	0.998	0.047
Catastrophic Illness	-	-	-	-	-	-	-	-	-	-	-
Without	148	63,571.83	232.81	562	192,669.45	291.69	0.798	0.806	0.672	0.958	0.020
With	20	19,473.89	102.70	71	61,358.72	115.71	0.888	0.899	0.758	1.094	0.182
Marital status	-	-	--	-	-	-	-	-	-	-	-
Without	70	38,033.11	184.05	295	120,005.45	245.82	0.749	0.754	0.632	0.891	<0.001
With	98	45,012.60	217.72	338	134,022.73	252.20	0.863	0.870	0.731	0.984	0.022
Education (years)	-	-	-	-	-	-	-	-	-	-	-
<12	88	38,948.45	225.94	320	122,940.53	260.29	0.868	0.867	0.721	0.979	0.023
≥12	80	44,097.26	181.42	313	131,087.65	238.77	0.760	0.765	0.634	0.910	<0.001
Season	-	-	-	-	-	-	-	-	-	-	-
Spring	38	18,725.29	202.93	155	57,375.11	270.15	0.751	0.759	0.632	0.902	0.001
Summer	39	20,940.50	186.24	165	65,150.71	253.26	0.735	0.743	0.621	0.880	<0.001
Autumn	40	25,007.28	159.95	162	72,600.25	223.14	0.717	0.724	0.608	0.863	<0.001
Winter	51	18,372.65	277.59	151	58,902.11	256.36	1.083	1.094	0.924	1.286	0.388
Urbanization level	-	-	-	-	-	-	-	-	-	-	-
1 (Highest)	42	24,936.20	168.43	182	79,857.63	227.91	0.739	0.742	0.611	0.897	<0.001
2	66	37,254.28	177.16	269	112,534.77	239.04	0.741	0.749	0.620	0.903	0.002
3	11	5966.48	184.36	36	18,479.15	194.81	0.946	0.953	0.798	1.138	0.265
4 (Lowest)	49	14,888.76	329.11	146	43,156.63	338.30	0.973	0.981	0.824	1.206	0.402
Level of care	-	-	-	-	-	-	-	-	-	-	-
Hospital center	54	31,037.93	173.98	214	97,437.34	219.63	0.792	0.799	0.635	0.950	0.009
Regional hospital	71	37,481.36	189.43	270	114,617.84	235.57	0.804	0.812	0.682	0.964	0.018
Local hospital	43	14,526.42	296.01	149	41,973.00	354.99	0.834	0.843	0.710	0.997	0.047

PYs—person-years; Adjusted HR—adjusted hazard ratio, adjusted for the variables listed in Table 3; CI—confidence interval.
